# Update on pellucid marginal degeneration

**DOI:** 10.1007/s00417-025-07022-1

**Published:** 2025-11-10

**Authors:** Ibraim V. Vieira, Victoria H. Fan, Charles Q. Yu

**Affiliations:** 1 Department of Ophthalmology, Byers Eye Institute at Stanford, 94303 Palo Alto, CA USA; 2 Department of Ophthalmology, Federal University of Sao Paulo, Sao Paulo, Brazil; 3 Stanford University School of Medicine, Stanford CA, USA

**Keywords:** Cornea, Ectasia, Pellucid, Keratoconus, Collagen, Crosslinking, Marginal, Degeneration

## Abstract

**Purpose:**

To review recent developments (2014–2024) in the diagnosis and treatment of pellucid marginal degeneration (PMD), focusing on advances in corneal imaging technologies, biomechanical assessments, and the effectiveness of current contact lens treatments and surgical techniques. Additionally, to discuss the role of PMD in cataract surgery and the use of toric intraocular lenses (IOLs).

**Method:**

Structured search of PubMed, Scopus, and Embase (Nov 2024; Jan 2014–present) using PMD-related keywords/MeSH. After dual independent screening of 129 citations using predefined inclusion/exclusion criteria, 50 articles met eligibility and were included.

**Results:**

Recent technologies enable better assessment of corneal curvature and pachymetry relationships, aiding differentiation of PMD from other ectatic diseases. Advances in biomechanical assessments have improved early detection and diagnostic precision. Most patients can be managed conservatively with new types of rigid contact lenses. Modified corneal cross-linking (CXL) protocols show promise in stabilizing the disease. Lamellar transplants offer visual outcomes comparable to penetrating keratoplasty with fewer complications. Wedge resection shows promising outcomes, but larger studies with long-term follow-up are still required. In cataract surgery, toric IOLs may provide effective astigmatism correction in selected PMD patients.

**Conclusion:**

The diagnosis and treatment of PMD have significantly evolved in the last decade. Improved imaging and biomechanical assessments aid in accurate diagnosis. Contact lenses and lamellar surgery are mainstay treatments. Future clinical studies should focus on the CXL protocols.

## Key Messages

*****What is known***:**

- Pellucid marginal degeneration is a corneal ectatic disorder of unknown cause.
- Cornea transplant has a high failure rate in pellucid marginal degeneration.

*****What is new***:**

- Contact lenses can manage most cases of mild to moderate pellucid marginal degeneration.
- Lamellar corneal surgery is useful in advanced cases of pellucid marginal degeneration. Collagen crosslinking may help prevent disease progression, but the optimal timing for treatment is still uncertain.

## Introduction

**Fig. 1:**
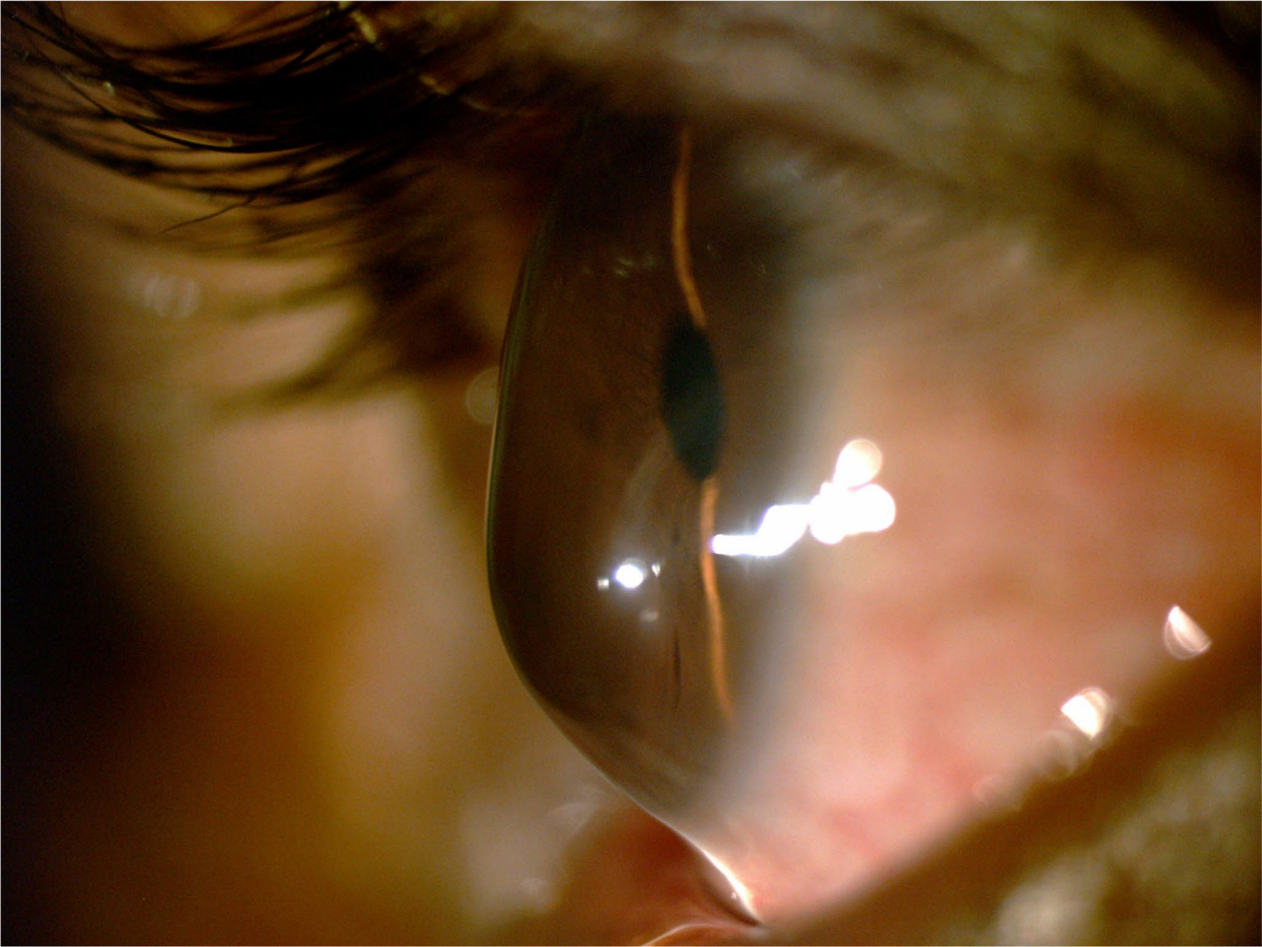
Appearance of advanced pellucid marginal degeneration

Pellucid marginal corneal degeneration (PMD) is an idiopathic, non-inflammatory thinning disorder of the peripheral cornea, typically affecting the inferior quadrant with corneal protrusion occurring above the area of thinning [1, 2] (Fig. 1). The condition is characterized by a gradual thinning of the inferior cornea, leading to significant visual impairment due to high irregular astigmatism. Although PMD is less common than keratoconus (KCN), it remains challenging for physicians to manage, as it is less frequently discussed in the literature and addressed in only a limited number of studies. Nevertheless, its impact on patients’ quality of life can be profound, highlighting the importance of timely diagnosis and appropriate management. This review provides an update on the latest findings in the diagnosis and treatment of PMD.

## Methodology - search strategy

A literature search was conducted in November 2024 across the Pubmed, Scopus, and Embase databases, covering the period from January 2014. The search utilized a combination of keywords and MeSH terms, including “pellucid marginal degeneration,” “PMD,” “diagnosis,” “treatment,” “topography,” “tomography,” “crosslinking,” “keratoplasty,” and “contact lens.” The initial search yielded 129 articles. Two authors independently screened titles and abstracts to identify potentially relevant publications. The full texts of these articles were then assessed for eligibility based on predefined criteria. Inclusion criteria were: articles published in English, clinical studies, case series with more than five patients, and comprehensive reviews relevant to clinicians. Exclusion criteria included single case reports (unless introducing a novel technique), letters to the editor, and non-English publications. After this review process, 50 articles were selected for inclusion in this review.

### Etiology and epidemiology

The exact etiology of PMD remains unknown, and its pathogenesis is not fully understood. Both KCN and PMD result from biomechanical fragility of the cornea, leading to progressive ectasia [3]. While KCN is associated with systemic diseases such as Down syndrome, connective tissue disorders, and atopic disease, there is no strong evidence linking PMD to systemic diseases [4]. Genetic factors may play a role, but the inheritance pattern of PMD is not established.

Although there is still some controversy, PMD **i**s probably more common in men than women [1, 5, 6]. Its prevalence is less than that of KCN, which has been estimated at approximately 1.38 per 1000 individuals [7]. However, the true prevalence of PMD may be underestimated due to misdiagnosis as KCN or other corneal ectatic disorders. Increased awareness and improved diagnostic techniques aid in accurate identification and management of PMD.

## Diagnosis

In PMD, corneal bulging occurs above the thinned region, whereas in KCN, maximal thinning coincides with the point of greatest protrusion [8]. PMD differs from other corneal thinning disorders, such as Mooren’s ulcer or Terrien’s marginal degeneration, as the area of thinning in PMD is epithelialized, clear, avascular, and without lipid deposition or significant inflammation.

Clear diagnostic guidelines or a classification system for PMD do not yet exist [8]. PMD is diagnosed based on a combination of clinical history, slit-lamp examination, and advanced imaging techniques such as Placido-based corneal topography, Scheimpflug tomography, and AS-OCT [9]. A comprehensive evaluation that incorporates these elements helps to accurately distinguish PMD from other ectatic diseases, with further details provided in the following sections.

## Clinical findings

Patients with PMD often present with longstanding poor vision or a slow, progressive reduction in visual acuity due to significant irregular astigmatism [6]. Visual symptoms may include blurred vision, distortion, ghosting, and halos around lights. Typically, PMD presents at an older age than KCN, with reported mean ages at presentation of 34 ± 15 years in some series [4] and as high as 48 ± 12 years in others [6].

Recently, Bari and Lyne [10] introduced the “inferior crescent sign” in PMD diagnosis, which is observed in advanced stages of the disease. It is characterized by a crescent-shaped illumination that appears above the inferior limbus when a penlight torch is shone from the superior quadrant. This was observed in five of nine cases they examined. Bhayana et al. [11] highlighted the utility of distant indirect ophthalmoscopy as a screening tool for PMD, reporting that a pear shaped red reflex was useful in a bedside diagnosis of PMD.

While PMD most commonly involves the inferior cornea, it can affect all quadrants [12, 13]. The condition is characterized by crescent-shaped stromal thinning of the peripheral cornea, with a band of thinning 1.0 to 2.0 mm wide, separated from the limbus by an uninvolved area of similar width [8]. Corneal protrusion is most pronounced just above the thinned region, leading to a “beer-belly” appearance when viewed in profile.

Severe thinning can lead to the loss of up to 80% of stromal tissue. Rarely, fluid entry into the corneal stroma following rupture of Descemet’s membrane can lead to acute hydrops, with subsequent vascularization and scarring [14]. Spontaneous perforation is rare but **does occur**, highlighting the importance of close monitoring in advanced cases.

## Distinction from keratoconus

Differentiating PMD from inferior KCN is crucial for determining the appropriate management. Mohr et al. [15] utilized wide-field spectral-domain OCT corneal sublayer pachymetry (RTVue XR) to differentiate PMD from KCN. They noted the ratio of corneal thickness in the inferotemporal 2–5 mm and in the inferior 7–9 mm sectors yield a high diagnostic accuracy for distinguishing between the two conditions.

Stachon et al. [16] reported different mRNA expression patterns in keratocytes of keratoglobus and PMD in comparison to KCN, suggesting distinct molecular profiles and underlying pathophysiological mechanisms. In the immunology field, Regueiro et al. [17] discussed toll-like receptors as potential diagnostic targets indicating that conjunctival TLR4 increased expression is identified as the most specific diagnostic marker for PMD. These results identify a possible immunological component in PMD pathogenesis and opens new avenues of research for the cause and treatment of this condition.

## Ancillary exams

### Topography

**Fig. 2:**
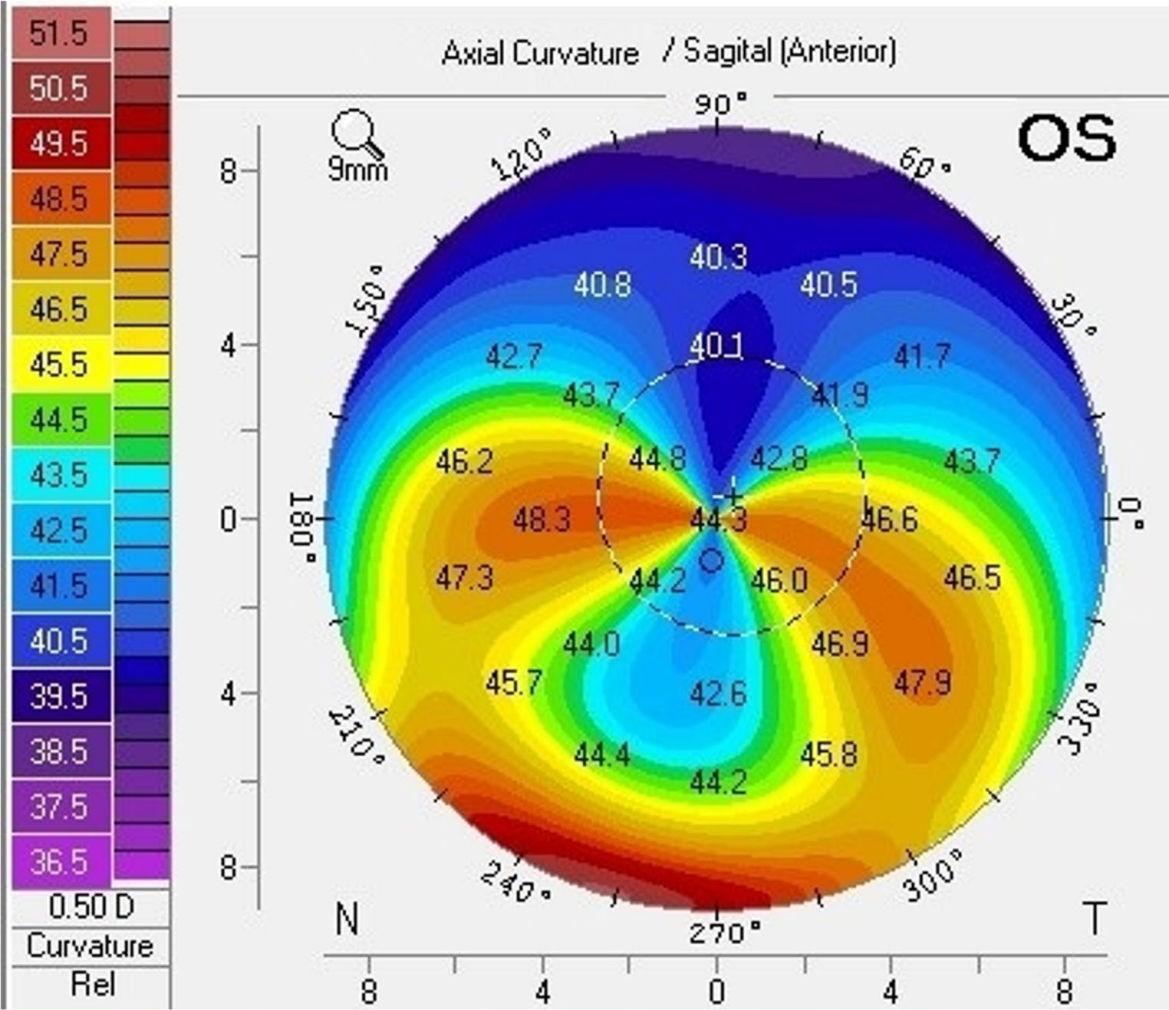
Characteristic kissing doves or crab claw appearance of pellucid marginal degeneration

Topographically, PMD presents as flattening along the central vertical axis and increased curvature in the inferior periphery, extending to the inferior oblique meridians, representing irregular astigmatism [18]. This pattern is often termed “crab claw” or “kissing doves.” (Fig. 2) However, similar patterns can appear in eyes with inferiorly decentered KCN, so diagnosis should consider clinical history, slit-lamp examination, and pachymetry maps alongside corneal topography. PMD typically presents later without atopy or habitual eye rubbing, shows a clear avascular crescent of inferior peripheral thinning with protrusion just above it, and on pachymetry an inferior band of thinning separated from the limbus with the thinnest point below the area of maximal curvature; KCN usually presents earlier with atopy/rubbing, central/paracentral cone signs (Fleischer ring, Vogt striae, apical scarring), and focal thinning at the cone apex.

### Tomography

**Fig. 3:**
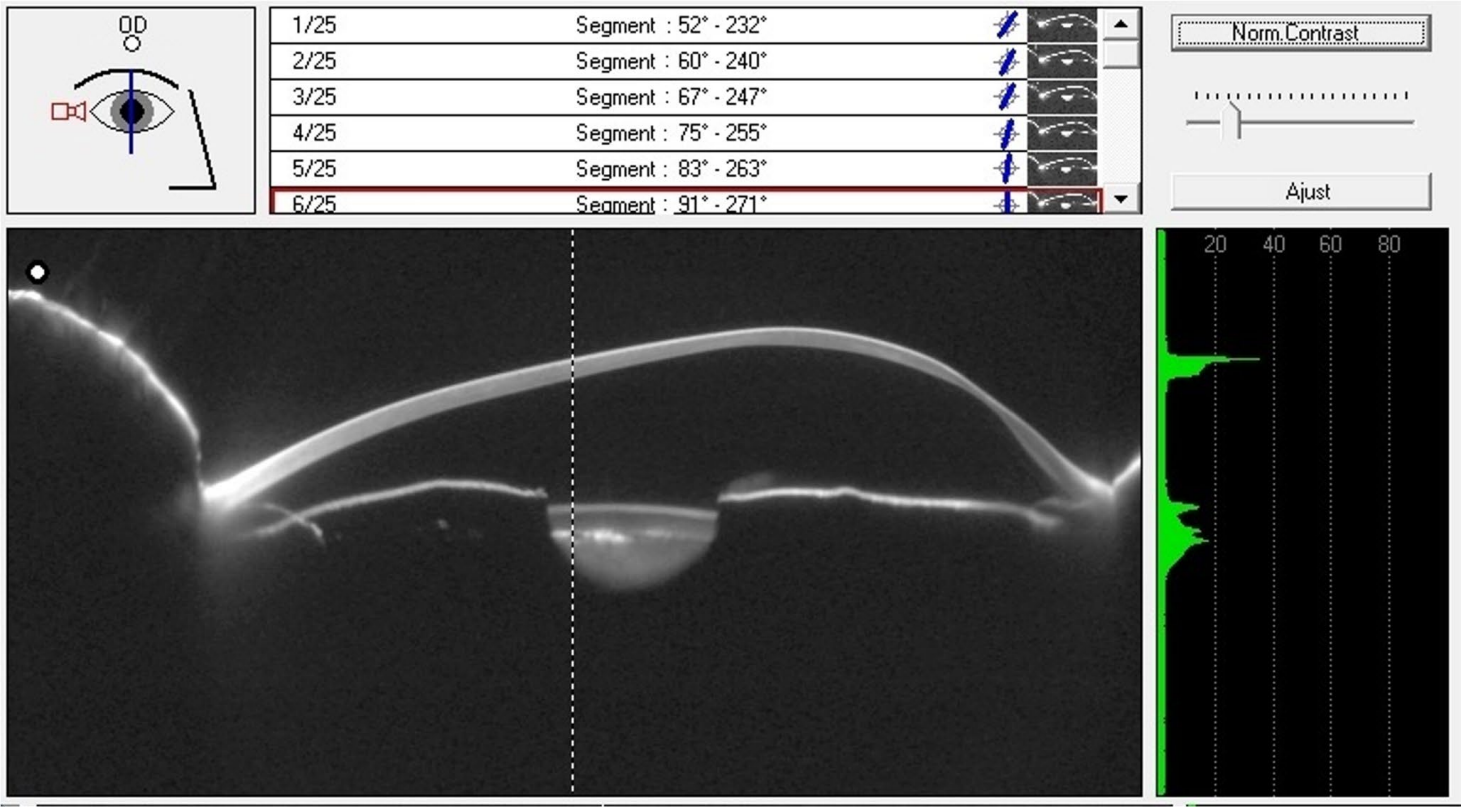
Thinning occurs below the point of maximal protrusion

Corneal tomography allows three-dimensional assessment of the cornea, correlating curvature alterations with thinning areas by producing both topographic and pachymetric maps. Devices like the Pentacam HR use Scheimpflug photography to acquire information on corneal topography, thickness, and posterior curvature [19]. PMD exams typically show thinning near the inferior limbus with bulging above it (Fig. 3).

 Beyond initial diagnosis, serial topography and tomography are invaluable for longitudinal monitoring of PMD. Progression can be documented by observing changes in key parameters, such as an increase in inferior corneal steepening, a decrease in minimum pachymetry, or alterations in posterior elevation maps over time. Such evidence of progression is a critical factor when considering interventions like corneal crosslinking [9, 19].

AS-OCT technologies, such as the Visante Omni system and RTVue Premier, have demonstrated high levels of reproducibility and agreement with tomographs and can be used for PMD assessment [20–22]. AS-OCT provides high-resolution cross-sectional images of the cornea, allowing precise measurement of corneal layers and detection of early changes in corneal architecture.

## Biomechanics

Identifying corneas with biomechanical fragility before ectasia onset has led to the development of in vivo biomechanical assessment technologies. Devices like the Ocular Response Analyzer (ORA) and Corvis ST evaluate corneal distensibility using air pulses and applanation response [23, 24]. These instruments provide parameters such as corneal hysteresis (CH) and corneal resistance factor (CRF), which reflect the viscoelastic properties of the cornea. Sedaghat et al. [25] compared ORA biomechanical indices among PMD patients, KCN patients, and normal controls, finding lower CH and CRF in PMD patients. However, sensitivity and specificity were insufficient for definitive clinical application. Labiris et al. [12] and Mergen et al. [26] also reported altered corneal biomechanics in PMD but concluded that established diagnostic tools remain primary, as biomechanical assessments currently lack the diagnostic precision needed for routine clinical use in PMD. Thus far hysteresis does not seem to be accurate enough for use in PMD diagnosis.

## Treatment

### Glasses

In early stages of PMD, spectacles can provide sufficient vision for daily activities. High astigmatism may cause vision oscillation and image distortion, and patients may require frequent prescription updates. Lenses with higher refractive indices can reduce eyeglass thickness and enhance cosmetic appeal, improving patient comfort and compliance.

### Contact lens

Although soft contact lenses may be used initially, rigid gas-permeable (RGP) contact lenses yield the best results for patients with high astigmatism and corneal irregularity. Tear-filling of the space between the cornea and the posterior surface of the lens eliminates anterior irregular astigmatism, greatly improving image quality. Challenges include inferior edge stand-off and unstable centration due to the peripheral corneal shape.

Scleral lenses, including the PROSE lens [27], which are supported by the sclera, offer better centration and comfort, improving visual quality and stability [28, 29]. They vault over the cornea, minimizing contact with the sensitive corneal surface and providing a smooth refractive surface. Scleral lens can significantly improve vision in PMD but require extensive training and patient dexterity to overcome placement difficulties. Proper patient education and follow-up are essential to ensure successful lens wear. Scleral lenses can also cause short term corneal changes in eyes with PMD, mostly anterior flattening due to edema [30].

Hybrid contact lenses, combining soft and rigid elements, are another option. The soft skirt improves comfort and centration, while the rigid center corrects astigmatism. For advanced disease or high astigmatism, bi-toric RGP lenses may be necessary to correct posterior astigmatism and refractive errors. Tzelikis et al. [6] retrospectively evaluated 85 eyes of 45 PMD patients, finding that most (88.2%) were managed non-surgically with spectacles or contact lenses. Only 11.8% underwent penetrating keratoplasty, indicating that a majority of patients can be treated conservatively. These findings underscore the great importance of RGP contact lens fitting in managing PMD.

### Surgical treatment

Various surgical procedures have been proposed for PMD, though many are limited to case reports and small series. In this review, we focus on the most recognized and commonly performed techniques.

### Collagen crosslinking (CXL)

CXL strengthens corneal collagen fibers, increasing resistance to deformation and halting disease progression. While standard protocols are established for KCN, their application in PMD is less clear due to the peripheral location of corneal thinning. Koller et al. [31] found that the OCT demarcation line in the corneal periphery is shallower, suggesting reduced efficacy in PMD. They proposed decentralized or more intense CXL to address this issue.

Modified CXL protocols, such as customized irradiation patterns and higher fluence settings, have shown promise in treating PMD. Pircher et al. [9] performed inferior decentered irradiation CXL on 16 PMD eyes, finding a small reduction in astigmatism and no evidence of progression over one year. They also observed an increase in inferior corneal thickness post-CXL, suggesting potential biomechanical strengthening. Irajpour et al. evaluated CXL in PMD over five years [32], concluding the technique is safe and granted stabilization is most patients. Still, he calls for larger studies to confirm efficacy. Irajpour et al. evaluated CXL in PMD over five years [32], concluding the technique is safe and granted stabilization in most patients.

Kymionis et al. [33] combined transepithelial photorefractive keratectomy (PRK) with CXL in PMD patients, achieving improved uncorrected distance visual acuity (UDVA) and reduced astigmatism without complications. This combined approach aims to regularize the corneal surface while stabilizing the ectasia. Nonetheless, concerns remain about tissue ablation in already thinned corneas, and the safety of such procedures in PMD requires further investigation.

Potential risks associated with CXL in PMD include corneal haze, delayed epithelial healing, and progression of ectasia if not properly tailored. De Almeida Ferreira et al. [34] reported cases of late progressive corneal flattening, haze, and visual loss after eccentric CXL for PMD, highlighting the need for caution and long-term monitoring. Collagen crosslinking must be performed prior to significant thinning of the cornea to prevent damage to endothelium because of treatment. It is unclear how much progression and when is the ideal time to perform collagen crosslinking in PMD. Based on the existing evidence in PMD and data extrapolated from other corneal ectatic disorders, the authors believe that CXL is likely most useful in early cases of PMD with documented progression.

### Corneal transplantation

#### Penetrating keratoplasty (PK)

PK may be considered for patients intolerant to lenses or with insufficient vision correction from contact lenses. Challenges include the peripheral thinning area, which complicates graft sizing and positioning. Large or decentered grafts increase rejection risk due to proximity to limbal and conjunctival vessels, and may lead to higher rates of astigmatism postoperatively. Other complications include stromal vascularization and acute rejection, edema related to viral infection, recurrent ectasia, and traumatic dehiscence. We reported a case of late spontaneous Descemet’s membrane detachment leading to corneal edema, highlighting the need to include this in the deferential for sudden corneal edema in a PMD patient who has had cornea transplant [35].

### Lamellar keratoplasty

Deep anterior lamellar keratoplasty (DALK) has emerged as an alternative to PK, offering reduced risks associated with open-globe surgery and endothelial rejection while providing comparable visual outcomes [36–38]. DALK preserves the recipient’s endothelium, reducing the risk of graft failure and allowing for larger grafts without increasing rejection risk. Al-Torbak [38] evaluated DALK in 16 PMD eyes, noting significant improvements in refractive outcomes and visual acuity without serious complications over an average follow-up of 14.6 months. Although peripheral thinning makes deep lamellar dissection technically demanding, DALK can be performed successfully in PMD, including advanced cases, in experienced hands. Intraoperative micro perforations may be managed without routine conversion to PK. The relative role of DALK versus alternative lamellar approaches is surgeon-dependent.

**Fig. 4:**
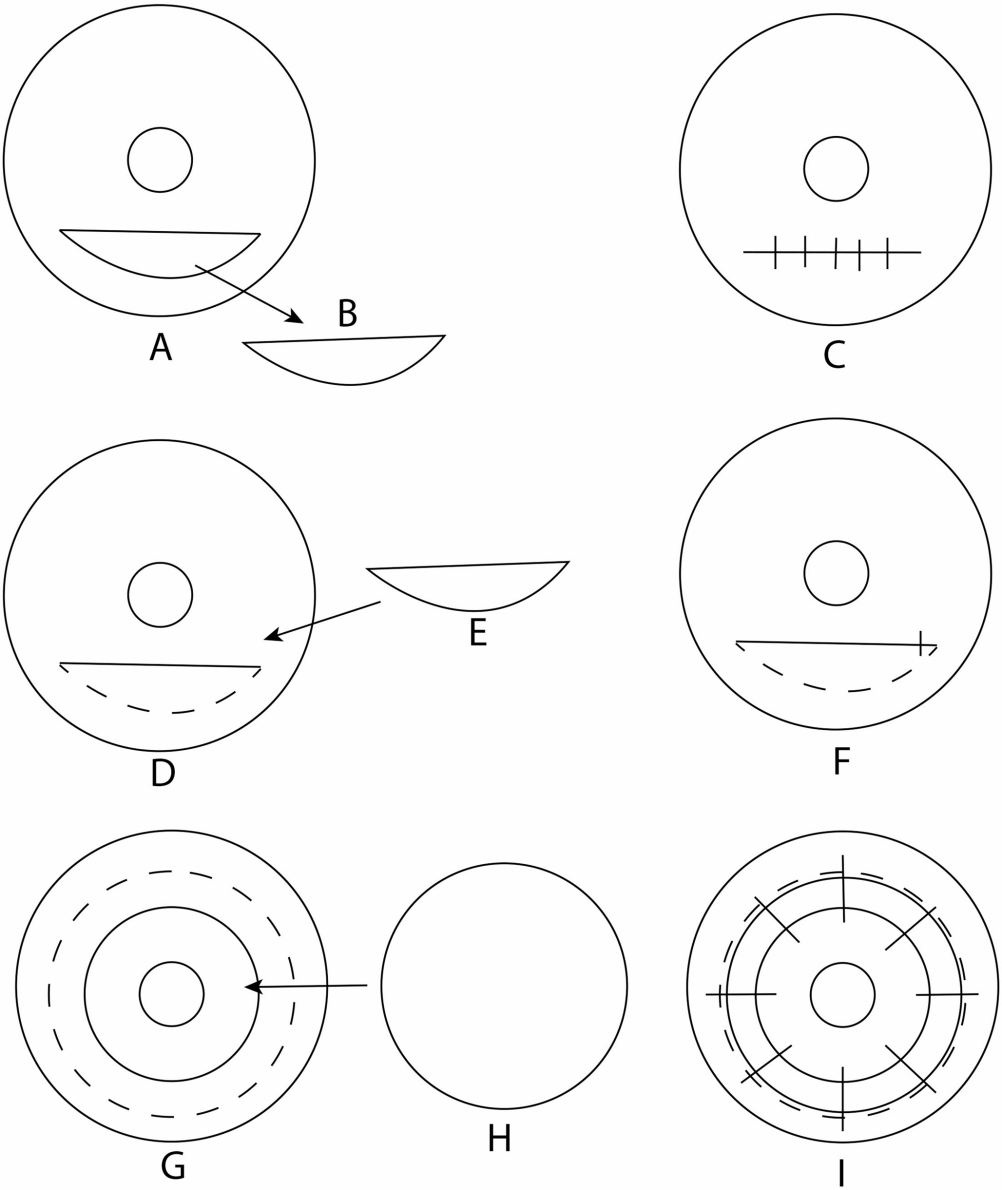
Advanced surgical techniques. In lamellar wedge dissection, a non-full thickness dissection (**B**) is excised from the inferior cornea (**A**). This is this closed with sutures (**C**). In intrastromal lamellar keratoplasty, a partial thickness pocket is dissected in the inferior cornea. (**D**) A full thickness (Jabbaravand et al.) or partial thickness (Gonçalves et al.) corneal tissue of appropriate shape is inserted. (**E, F**) A single suture is used to close the pocket. In tuck in lamellar keratoplasty, a central partial thickness trephination is done and a circular central layer of cornea is removed. The edge of the wound is then dissected in a lamellar manner towards the limbus (**G**). A circular donor cornea without endothelium (**H**) is then placed through the central incision and tucked into the lamellar dissection and secured with sutures (**I**)

Other techniques include crescentic lamellar keratoplasty and intrastromal lamellar keratoplasty. Jabbarvand et al. [39] performed intrastromal lamellar keratoplasty in 10 PMD patients, finding improvements in UDVA and CDVA. The procedure involves implanting a donor stromal lenticule into a pocket in the peripheral cornea, aiming to reinforce the thinned area without full-thickness grafting. (Fig. 4D, F) Vajpayee et al. [40] introduced the “tuck-in” lamellar keratoplasty (TILK), showing significant vision improvement and reduced astigmatism. In TILK, a donor corneal button is sutured into a peripheral stromal pocket, providing structural support and improving corneal curvature. (Fig. 4G, I) Gonçalves et al. [41] described partial-thickness intrastromal lamellar keratoplasty for PMD, demonstrating its potential as a less invasive option with fewer complications. (Fig. 4D, F**) **However, these surgeries all involve lamellar dissection of already thin cornea tissue like in DALK, and with similar risk of intraoperative perforation requiring PK.

### Lamellar wedge resection

Wedge resection involves removing the affected corneal segment and suturing the edges to reduce astigmatism and normalize corneal curvature. (Fig. 4A, C) It can be performed while preserving the endothelium, reducing intraocular risks. The procedure is relatively simple and cost-effective, making it accessible in regions with limited resources. Genç et al. [42] evaluated lamellar wedge resection in 10 PMD eyes, finding significant improvements in UDVA and BCVA and a reduction in astigmatism over an average follow-up of 14.1 months. Maccheron and Daya [43] reported similar findings, suggesting that wedge resection is a viable alternative, especially where donor corneas are scarce. Kymionis et al. [44] combined corneal wedge resection with CXL for PMD in a case report, presenting positive outcomes and proposing that the combination may enhance stability and visual results. Recently, Michael et al. [45] introduced an endothelium-sparing, air-assisted wedge resection technique also presented in a case report, emphasizing safety, simplicity, and efficacy.

### Intrastromal corneal ring segment (ICRS)

ICRS implantation is a reversible procedure used in moderate to advanced KCN which has also been explored in PMD. The rings add tissue to the middle periphery, flattening the central cornea and reducing irregular astigmatism by altering corneal biomechanics. There are currently several models of ICSRS, which differ from one other in terms of their material, thickness, width or arc length.

Jabbarvand et al. [46] reported on the implantation of MyoRing segments in 33 PMD eyes, noting improvements in UDVA, mean keratometry, and refractive errors without significant complications. Ertan and Bahadır [47] evaluated Intacs ring segments in nine PMD eyes, finding significant improvements in uncorrected and corrected visual acuity and cylindrical refraction.

Hashemian et al. [48] studied outcomes of single-segment ICRS in early PMD, demonstrating its potential effectiveness. In advanced disease, however, marked irregular astigmatism and pronounced peripheral thinning often preclude safe implantation and heighten the risk of complications (malposition/extrusion, corneal perforation). Additional ICRS-related issues include regression, infection, glare/halos, and subsequent difficulty with contact-lens fitting. When ectasia is progressive, CXL is often performed before or combined with ICRS, but the implant does not address the characteristic peripheral thinning or progression by itself.

 A newer technique, Corneal Allogenic Intrastromal Ring Segments (CAIRS), utilizes donor corneal tissue instead of synthetic material. While not yet extensively studied in PMD, it represents an emerging area of interest for corneal regularization [49].

### Cataract surgery and PMD

Cataract surgery in PMD patients poses significant challenges due to irregular astigmatism and altered corneal biomechanics. Surgical planning should include careful consideration of incision placement. While clear corneal incisions may be performed, a scleral tunnel incision may be considered to minimize impact on corneal astigmatism, particularly in eyes with extreme peripheral thinning where incision architecture could be compromised.

Accurate biometry and IOL power calculation can be difficult, potentially leading to refractive surprises postoperatively. Furthermore, the implantation of toric intraocular lenses (IOLs) should be considered primarily for patients who have not achieved satisfactory adaptation to RGP contact lenses. Considering that RGP lenses effectively correct corneal astigmatism, if a patient continues to use contact lenses postoperatively, any residual lenticular astigmatism will remain uncorrected, potentially compromising visual outcomes.

Until recently, the use of toric IOLs could correct regular astigmatism, but irregular astigmatism would persist. Recently Paryani et al. [50] reported a case on the aberrometric outcomes of customized toric IOL implantation in a cataract patient with high corneal astigmatism due to PMD. They found that customized toric IOLs could effectively reduce astigmatism and improve visual quality. While access to such customized IOLs may be limited and is often part of clinical studies, it highlights a potential future direction for these complex cases.

Preoperative evaluation should include detailed corneal topography and tomography to assess the regularity and magnitude of anterior and posterior corneal astigmatism. Intraoperative aberrometry may aid in refining IOL power calculation and axis alignment. The use of toric IOLs in PMD patients is promising but requires careful patient selection, informed consent, and surgical planning. Further studies are needed to establish guidelines and optimize outcomes for toric IOL implantation in this population.

## Conclusion

Although the underlying etiology of PMD remains poorly defined, clinical management has advanced substantially in the past decade. Ongoing research in genetics and corneal biomechanics may enable earlier diagnosis and the development of targeted therapies.

Current practice favors conservative, cornea-preserving approaches supported by advances in corneal tomography and modern therapeutic options. Most patients are successfully managed with scleral or hybrid lenses, while corneal cross-linking is applied to halt documented progression—though randomized longitudinal studies are still needed to establish optimal timing and patient selection.

For advanced disease, endothelium-sparing procedures—including lamellar wedge resection, ICRS, DALK, and its variations—represent viable surgical alternatives. The choice typically reflects surgeon expertise and corneal anatomy, with no evidence of superiority among them when the endothelium is preserved. PK is generally reserved for complex or refractory cases.
